# Aging Abroad: Do Eastern Europeans Experience the Healthy Immigrant Effect?

**DOI:** 10.1093/geroni/igaf122.3415

**Published:** 2025-12-31

**Authors:** Adriana Scanteianu

**Affiliations:** University of Pennsylvania, Philadelphia, Pennsylvania, United States

## Abstract

As the European continent continues to undergo population aging, there is a growing number of migrants who are also aging alongside the native population. Immigrants occupy a unique socioeconomic position and often face health risks and care access issues that are poorly understood, yet critical to informing policy and facilitating healthy aging. In particular, post-Communist Eastern European migration has received little attention in the public health literature despite constituting a sizable immigrant population in Western Europe and Israel. Furthermore, existing literature points to Eastern Europeans as potentially presenting an exception to the “healthy immigrant effect,” whereby immigrants usually experience healthier and longer lives than native-born populations despite often having relatively lower socioeconomic status and lacking institutional support. Using data from the Survey of Health, Aging, and Retirement in Europe (SHARE), which provides longitudinal coverage of all EU countries and Israel, this study aims to understand whether Eastern European migrants in Western Europe and Israel experience a health disadvantage compared to other migrant groups and the native-born populations. Findings reveal stark disparities in health outcomes between Eastern European migrants when compared to other migrant groups, as well as the native-born populations in Western Europe and Israel, including differences in mental health, disability, and chronic disease prevalence. These results have strong policy implications, as they imply distinct health and social support needs for the Eastern European migrant population in receiving countries.

